# A role for TSPO in mitochondrial Ca^2+^ homeostasis and redox stress signaling

**DOI:** 10.1038/cddis.2017.186

**Published:** 2017-06-22

**Authors:** Jemma Gatliff, Daniel A East, Aarti Singh, Maria Soledad Alvarez, Michele Frison, Ivana Matic, Caterina Ferraina, Natalie Sampson, Federico Turkheimer, Michelangelo Campanella

**Affiliations:** 1 Department of Comparative Biomedical Sciences, The Royal Veterinary College, University of London, Royal College Street, London NW1 0TU, UK; 2 Regina Elena-National Cancer Institute, 00144 Rome, Italy; 3 Department of Neuroimaging, Institute of Psychiatry, King's College London, London, UK; 4 Department of Biology, University of Rome ‘TorVergata’, 00133 Rome, Italy; 5 Division of Experimental Urology, Medical University of Innsbruck, A6020 Innsbruck, Austria; 6 University College London Consortium for Mitochondrial Research, Gower Street, WC1E 6BT London, UK

## Abstract

The 18 kDa translocator protein TSPO localizes on the outer mitochondrial membrane (OMM). Systematically overexpressed at sites of neuroinflammation it is adopted as a biomarker of brain conditions. TSPO inhibits the autophagic removal of mitochondria by limiting PARK2-mediated mitochondrial ubiquitination via a peri-organelle accumulation of reactive oxygen species (ROS). Here we describe that TSPO deregulates mitochondrial Ca^2+^ signaling leading to a parallel increase in the cytosolic Ca^2+^ pools that activate the Ca^2+^-dependent NADPH oxidase (NOX) thereby increasing ROS. The inhibition of mitochondrial Ca^2+^ uptake by TSPO is a consequence of the phosphorylation of the voltage-dependent anion channel (VDAC1) by the protein kinase A (PKA), which is recruited to the mitochondria, in complex with the Acyl-CoA binding domain containing 3 (ACBD3). Notably, the neurotransmitter glutamate, which contributes neuronal toxicity in age-dependent conditions, triggers this TSPO-dependent mechanism of cell signaling leading to cellular demise. TSPO is therefore proposed as a novel OMM-based pathway to control intracellular Ca^2+^ dynamics and redox transients in neuronal cytotoxicity.

TSPO initially characterized as the peripheral binding site for the benzodiazepines^1, 2^ complexes with the voltage-dependent anion channel (VDAC).^3^ Even though questioned, the role of TSPO in transporting cholesterol into mitochondria for steroidogenesis^4, 5, 6^ remains the best-characterized one.^7^

TSPO, which is ubiquitously expressed throughout the peripheral tissues although at various levels^8^ is almost absent in healthy adult brain,^9^ to instead accumulate in age-related degenerative disorders^10^ and traumatic stress.^11^

Binding of TSPO is largely developed in diagnostic studies of neuroinflammation following successful protocols of positive emission tomography (PET).^12^ TSPO is therefore amenable to noninvasive diagnostic routines as well as to chemical targeting, thus making pivotal a comprehensive characterization of its molecular physiology for broader applications.

The binding of TSPO to VDAC, which is the principal Ca^2+^ channel of the OMM,^13^ could imply modifications in the homeostasis of this second messenger that contributes the inflammasome.^14^ Previous reports have shown that TSPO ligands modulate mitochondrial and cytosolic Ca^2+^ dynamics^15, 16^ as well as that TSPO itself impairs cellular mitophagy^17^ questioning whether it has a direct or indirect role in mitochondrial and cell signaling regulation. TSPO interacts with the Acyl-CoA binding domain containing 3 protein (ACBD3)^18^ that complexes with the cAMP-dependent protein kinase (PKA)^19^ which, via phosphorylation,^20^ regulates VDAC activity.

We have therefore formulated a working hypothesis whereby the overexpression of TSPO, induced during cellular stress, may be functionally relevant to Ca^2+^ homeostasis and cell signaling by anchoring the ACBD3/PKA complex on the mitochondria and therefore primes VDAC post-translational modifications.

Here, we have tested this using various approaches to finally propose a model, which sees TSPO as an inducible, OMM-based conduit, to control intracellular Ca^2+^ dynamics, redox transients and cytotoxicity.

## Results

### The Ca^2+^-dependent NADPH oxidase drives TSPO-associated ROS

**Figure 1:**
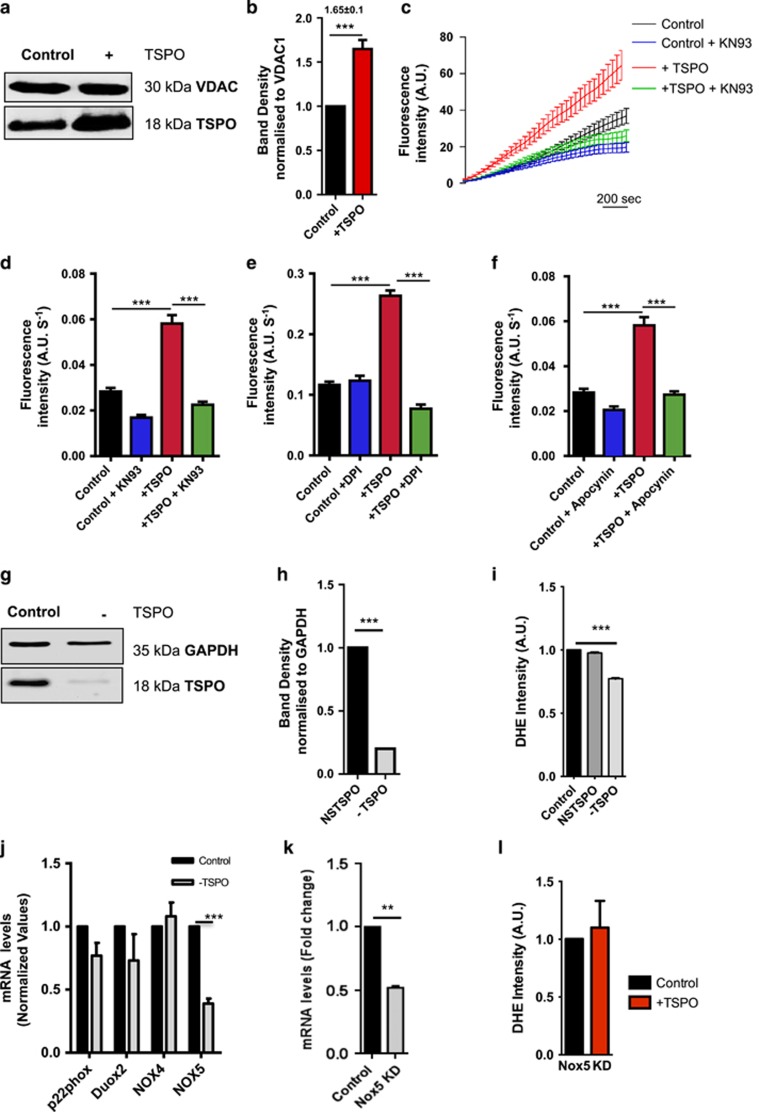
TSPO-associated ROS is derived from Ca^2+^-dependent NADPH oxidase. (**a**) Western blot showing TSPO overexpression in SH-SY5Y. (**b**) Quantification of TSPO overexpression. (**c**) Representative dihydroethidium (DHE) traces of TSPO knockdown SH-SY5Y cells in the presence of KN93. (**d**) Corresponding bar chart showing mean rate of increase in dihydroethidium fluorescence intensity; *n*=3 (⩾30 cells per field of analysis); *P*<0.001. (**e**) Bar chart showing the rate of increase in DHE fluorescence intensity in +TSPO cells treated with DPI (200 nM). (**f**) Bar chart showing the rate of increase in dihyroethidum fluorescence intensity in +TSPO cells treated with Apocynin (200 nM). (**g**) Western blot showing TSPO knockdown in SH-SY5Y cells (*n*=4, ⩾50 cells per field of analysis, *P*<0.001); (**h**) quantification of TSPO expression in SH-SY5Y cells (*n*=2, ⩾50 cells per field of analysis, *P*<0.001). (**i**) Bar chart showing the rate of increase in DHE fluorescence intensity in −TSPO cells (*n*=2, ⩾50 cells per field of analysis, *P*<0.001). (**j**) qRT-PCR studies of NOX isoforms in SH-SY5Y (*P*<0.001). (**k**) qRT-PCR study of NOX expression in control and NOX5 KD cells showing fold change in gene expression (*n*=3, *P*<0.001). (**l**) Bar chart showing the rate of increase in DHE fluorescence intensity in NOX5 KD cells (*n*=3, ⩾40 cells per field of analysis, *P*>0.05; **P<0.01, ***P<0.001)

We previously ascribed the inhibition of cellular mitophagy by TSPO to ROS microdomains that prevent the *in situ* ubiquitination for autophagic removal.^17^ The increased accumulation of cytosolic ROS following overexpression of TSPO – previously reported^17^ – was here confirmed in the neuronal cell line SH-SY5Y overexpressing TSPO (+TSPO; Figure 1a depicts transient overexpression of the protein with densitometry analysis reported in Figure 1b, MOCK-transfected cells (hereafter referred to as Control unless otherwise stated): 1.00±0.00; +TSPO: 1.65±0.1).

The increased ROS levels were reduced by KN93 (10 *μ*M), an inhibitor of the Ca^2+^/Calmodulin-dependent protein kinase (CaMK) II^21^ which is pivotal for the activation of the NADPH oxidase (NOX)^22, 23^ (traces reported in Figure 1c and rate of ROS increase in Figure 1d Control: 0.028±0.008; +TSPO: 0.058±0.003; Control +KN93: 0.017±0.007; +TSPO +KN93: 0.022±0.001). This implied events occurring downstream of the mobilization of intracellular Ca^2+^, which were therefore inferred pharmacologically by repeating the analysis in the presence of the specific NOX inhibitors DPI (diphenyleneiodonium) and Apocynin (200 nM)^24^ (Figure 1e and f).

Both inhibitors, consistent with the KN93 data, lowered cytosolic ROS levels in +TSPO conditions thus implying a link with the Ca^2+^-dependent NOX-originated ROS (DPI, Control: 0.11±0.03; +TSPO: 0.26±0.04; Control +DPI: 0.12±0.05; +TSPO +DPI: 0.08±0.0; Apocynin, Control: 0.028±0.008; +TSPO: 0.058±0.003; Control +Apocynin: 0.018±0.008; +TSPO +Apocynin: 0.027±0.009). The NOX family of superoxide (O_2_^•−^) and hydrogen peroxide (H_2_O_2_) producing proteins are indeed an important source of ROS in signal transduction.^25^

NOX proteins are composed of various isoforms, of which the isoform 5 is activated by Ca^2+^ and Calmodulin-based mechanisms^26, 27^ as it contains EF-hand Ca^2+^-binding domains^28, 29^ and therefore being sensitive to increases in the [Ca^2+^]_i_. Interestingly, TSPO has been recently proposed to interplay with NOX proteins.^30^ To ascertain this and corroborate the pharmacological evidences implying a correlation between TSPO, Ca^2+^ and ROS producing NOX proteins, we generated a SH-SY5Y cell line stably downregulated for TSPO (SH-TSPOkd hereafter referred to as −TSPO; protein expression levels, Control: 1.00±0.00; −TSPO: 0.016±0.004; Figures 1g, h, Supplementary Figures 1a and b), which had markedly reduced levels of ROS if compared with control (Control: 1; non-silencing (NSC): 0.99±0.004; −TSPO: 0.078±0.03; Figure 1i). Notably, the mRNA analysis of NOX isoforms revealed a significant, specific, reduction in the expression of the isoform 5 (Figure 1j; −TSPO, p22phox: 0.77±0.021 NOX2/DUOX2: 0.73±0.0021 NOX4: 1.08±0.001 NOX5: 0.39±0.004).

To validate this further, we created a SH-SY5Y cell line stably downregulated for NOX5 (Figure 1k; mRNA, Control: 1.±0.3; −NOX5: 0.52±0.1) in which we measured ROS levels following recombinant overexpression of TSPO that did not increase above control levels (Figure 1l; DHE rate of increase, Control: 1; −NOX5: 1.1±0.29). Having recorded that the TSPO-mediated accumulation of ROS is dependent on NOX signaling and likely via the Ca^2+^-dependent isoform, we thoroughly investigated the Ca^2+^ homeostasis.

### TSPO alters Ca^2+^ dynamics mammalian cells

**Figure 2:**
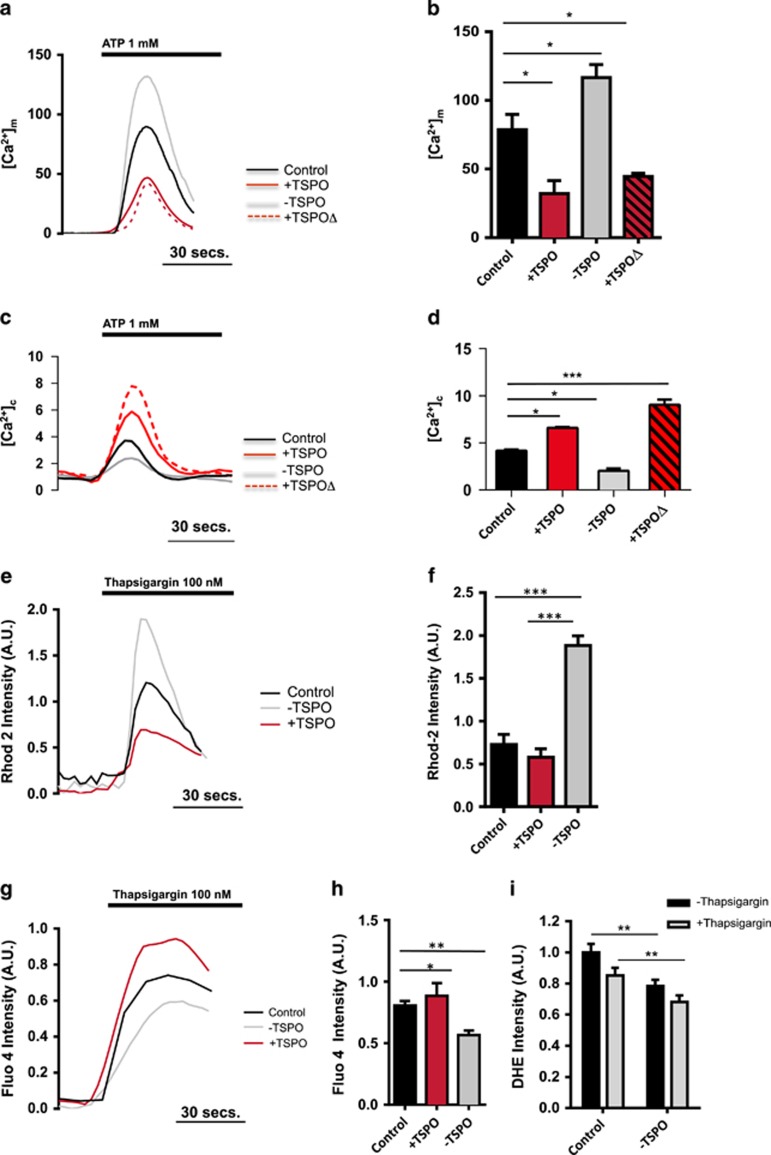
TSPO re-routes cellular Ca^2+^ signals in mammalian cells. (**a**) Representative Ca^2+^ transients from MEFs transfected with mtAeq and stimulated with ATP (1 *μ*M). (**b**) Graph showing the mean max [Ca^2+^]_m_ *n*=5; ⩾50 cells per field of analysis; *P*<0.05. (**c**) Representative Ca^2+^ transients from MEFs transfected with cytAeq and stimulated with ATP (1 mM). (**d**) Graph showing the mean max [Ca^2+^]_c_ *n*=3; ⩾50 cells per field of analysis; *P*<0.05. (**e**) Representative mitochondrial Ca^2+^ traces obtained in MEFs loaded with Rhod-2 and stimulated with Thapsigargin (100 nM). (**f**) Graph showing mean maximum Rhod-2 fluorescence intensity in MEFs. *n*=4; ⩾40 cells per field of analysis; *P*<0.001. (**g**) Representative cytosolic Ca^2+^ traces obtained in CF35 cells loaded with Fluo-4 and stimulated with Thapsigargin (100 nM). (**h**) Graph showing mean maximum Fluo-4 fluorescence intensity in MEFs (*n*=3; ⩾40 cells per field of analysis; *P*<0.001). (**i**) Mean intensity of DHE fluorescence between control and TSPO-KD cells treated with Thapsigargin (*P<0.05, **P<0.01, ***P<0.001)

To investigate the role of TSPO in Ca^2+^ signaling, MEFs with silenced TSPO were co-transfected with mitochondria-targeted aequorin (mtAEQ) – a Ca^2+^-sensitive photoprotein^31^ and perfused with ATP (1 mM). The resulting Ca^2+^ response was calibrated to quantify [Ca^2+^]_m_,^31^ which resulted which resulted in elevated levels of Ca^2+^ in TSPO knockdown (−TSPO) MEFs (Figure 2a), with a mitochondrial [Ca^2+^]_max_ of 132.2±9.4 compared with Control values of 89.78±12.2 (Figure 2b). Non-silencing TSPO shRNA (NSTPSO) was tested too and compared with Control and mtAEQ solely expressing MEFs (Supplementary Figure 1c). We also assessed the effects in wild-type TSPO-overexpressing cells (+TSPO) or expressing the mutant isoform of TSPO (+TSPOΔ) bearing a deletion of the cholesterol binding motif.^32^ In both these conditions, [Ca^2+^]_m_ was significantly impaired, indicating that TSPO-mediated Ca^2+^ regulation is independent from the cholesterol binding function (+TSPO: 32.10±9.4; +TSPOΔ: 46.97±2.36; Figures 2a and b). Comparable results were obtained in HeLa (Supplementary Figures 1d and e) and canine epithelial cells (CF35; Supplementary Figures 2a and b), a microglia cell line (BV2) and primary rat microglia cultures (Supplementary Figures 2f–h) suggesting a conserved regulatory mechanism. The differences observed in mitochondrial Ca^2+^ handling were also consistent with changes in the mitochondrial membrane potential (ΔΨ_m_) recorded in −TSPO cells (Supplementary Figures 3a and b).

We therefore assessed the cytoplasmic Ca^2+^ transients, using MEFs transfected with cytoplasmic-targeted aequorin (cytAEQ)^33^ reporting that the [Ca^2+^]_c_ was decreased in −TSPO cells (2.43±0.25) compared with Control (4.31±0.14), while significantly higher transients were observed in both +TSPO (6.70±0.10) and +TSPOΔ cells (9.58±0.55; Figure 2d). Also in this case, reciprocity of results was obtained in HeLa (Supplementary Figures 1f and g) and CF35 cells (Supplementary Figures 2c–e).

A well-established factor in the Ca^2+^ homeostasis is that alterations in the mitochondrial Ca^2+^ buffering capacity would subsequently shape the amplitude of the cytosolic signal.^31, 32^ To confirm this specific correlation also in cells expressing alternative TSPO levels, we used thapsigargin (Tg),^34^ which inhibits the sarco-endoplasmic reticulum Ca^2+^-ATPase (SERCA), leading to an IP_3_ independent rise in [Ca^2+^]_c_ through proton-leakage channels.^35^ Using a fluorescence-based approach, we found that in −TSPO cells Tg elicited a significantly greater rise in mitochondrial Ca^2+^ level as determined by Rhod-2 F_max_ (1.89±0.11) compared with the Control (0.73±0.12) and +TSPO (0.58±0.10; Figures 2e and f).

The same experiment performed in the cytosolic compartment demonstrated that overexpression of TSPO led to increased [Ca^2+^]_c_ (0.89±0.08) compared with Control (0.81±0.04), whereas TSPO knockdown restricted this rise (0.57±0.04; Figures 2g and h). TSPO may indeed control mitochondrial Ca^2+^ uptake rather than influencing the levels of Ca^2+^ stored in the ER or Ca^2+^ influx across the plasma membrane, thus influencing the tonicity of the cytosolic Ca^2+^ signals and the generation of ROS. The rate of ROS accumulation in SH-SY5Y cells is thus reduced, following depletion of Ca^2+^ from the intracellular stores via treatment with Tg and reduced further by TSPO deletion (Control: 1.03±0.05 Control Tg: 0.85±0.05 −TSPO: 0.78±0.04−TSPO Tg: 0.68± 0.04; Figure 2i).

### TSPO alters mitochondrial network morphology but not the ER-mitochondria connectivity

**Figure 3:**
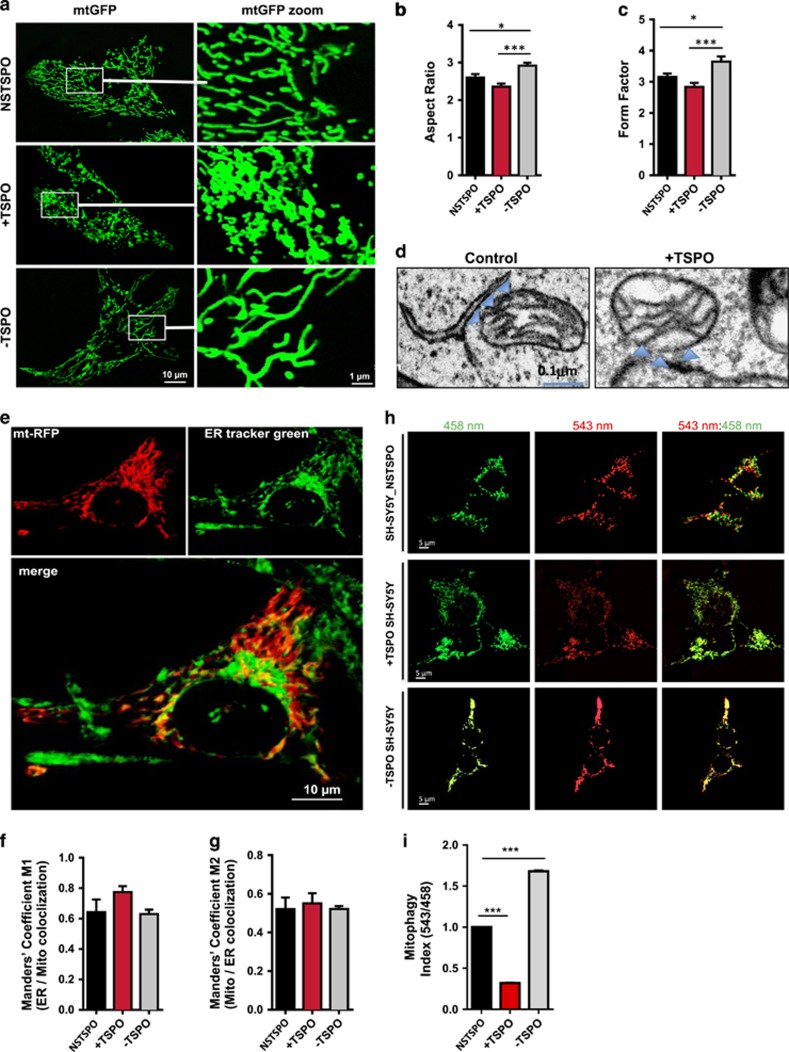
TSPO alters mitochondrial network morphology but not ER-mitochondria connectivity. (**a**) Representative mid-plane cross-sectional images of the cellular mitochondrial network obtained through confocal microscopy are depicted in the left panel. White boxes show regions of the cell that are then magnified in the right panel. (**b**) Bar chart showing the mean aspect ratio of mitochondria according to TSPO expression (*n*>15 cells; *P*<0.001). (**c**) Bar chart showing the mean form factor for mitochondria according to TSPO expression; *n*>15 cells; *P*<0.001. (**d**) Electromicrographs showing ER-mitochondria adjacency (**e**) Representative confocal image of which the green signal is indicative of ER-green and the red signal represents the mitochondrial network. Areas of mitochondria that overlap with the ER are colored yellow. (**f**) Manders’ coefficient M1, calculated by volume of ER in contact with mitochondria/ total volume of ER; *n*>5 cells; *P*>0.05. (**g**) Graph showing how the Manders’ coefficient M2, calculated by volume of mitochondria in contact with ER/total volume of mitochondria, varies according to TSPO expression. Graph shows the % mitochondria connected with the ER; *n*>5 cells; *P*>0.05. (**h**) Panel depicts mitoKeima measurement of mitophagy repression in SH-SY5Y cells expressing TSPO in alternative amount. (**i**) Mitophagy Index expressed as mitoKeima fluorescence ratio is quantified (*P<0.05, ***P<0.001)

Alterations in mitochondrial Ca^2+^ uptake pathways may be linked to morphological changes in the mitochondrial network. We therefore scrutinized mitochondrial morphology by analyzing images from cells expressing mitochondrial-targeted GFP, (mtGFP;^17^ Figure 3a). The mitochondrial aspect ratio, a measure of length and form factor and the degree of branching, revealed a tendency for the mitochondrial network in −TSPO cells to be longer (Figure 3b) and more branched (Figure 3c) than NSTSPO (which was used as Control as not bearing alterations, please see Supplementary Figure 3c), while shorter and less-branched networks were observed in +TSPO cells, consistent with our analysis on TSPO and Ca^2+^ handling. We have previously shown that TSPO expression level does not correlate to the levels of the pro-fission and pro-fusion proteins,^17^ although activation of DRP1 can be Ca^2+^-dependent.^36^

There is a well-established structural and functional coupling between the ER and mitochondrial network^37^ in which VDACs are found at higher densities^38^ and they are involved in ER-mitochondria tethering promoting mitochondrial Ca^2+^ uptake.^39^ We therefore explored whether altering TSPO/VDAC expression ratio may impact upon ER-mitochondria connectivity. Electron Micrograph evaluation of the adjacency between ER and mitochondria (Figure 3d) indicated that any TSPO-mediated alteration of Ca^2+^ signaling is independent from ER-mitochondria physical coupling. To corroborate this further, however, three-dimensional images were generated from cells double stained for the ER (Green, using ER Tracker Green) and mitochondria (Red, using the mtRFP; Figure 3e) thus allowing the determination of the coefficients of co-localization and overlap between the two signals.^40^ In the M1 cohort (indicating the fraction of the ER that co-localizes with mitochondria), TSPO expression level did not alter the level of interaction between mitochondria and ER (Figure 3f). There was a marginal increase in the +TSPO condition (0.77±0.04) compared with both NSTPO (0.64±0.08) and −TSPO (0.63±0.03) but this was not statistically significant (Figure 3f). Similarly, in the M2 cohort (indicating the fraction of mitochondria that co-localizes with the ER), TSPO expression level did not yield any significant difference (NSTPO: 0.52±0.06; +TSPO: 0.55±0.05; −TSPO: 0.52 ±0.01; Figure 3g). Increased TSPO/VDAC1 ratio inactivates the PARK2-mediated ubiquitination of mitochondrial proteins and the execution of mitophagy.^17^ Representative micrographs of mitophagy were acquired via confocal microscopy using the dual emission of the ratiometric pH-sensitive fluorescent protein mt-Keima^41^ in neuronal cells (SH-SY5Y) exposed to FCCP (1 *μ*M; Figure 3h). High ratio 543 nm/458 nm fluorescence ratio indicates mitochondria in acidic lysosomes resulting from their autophagic removal. As quantified in Figure 3i, this mitophagy index is reduced in TSPO-overexpressing cells (NSTPO: 1; +TSPO: 0.32±0.001, *n*=2), while greatly increased when the protein is ablated (NSTSPO: 1; −TSPO: 1.65±0.003, *n*=2). Also in this case, NSTSPO was used as control (Supplementary Figure 3d).

Furthermore, in VDAC1 knockout MEFs (VDAC1^−/−^), the mitochondrial network appears larger (Supplementary Figure 3e) similar to what is observed during TSPO overexpression confirming that, it is the TSPO ratio with VDAC1 the core variable in defining mitochondrial structure and function.

### TSPO-dependent regulation of Ca^2+^ is via VDAC1 and the PKA

The results on ER-mitochondria contact sites led us to investigate alternative regulatory pathways via which TSPO may impinge on intercellular Ca^2+^ signaling.

**Figure 4:**
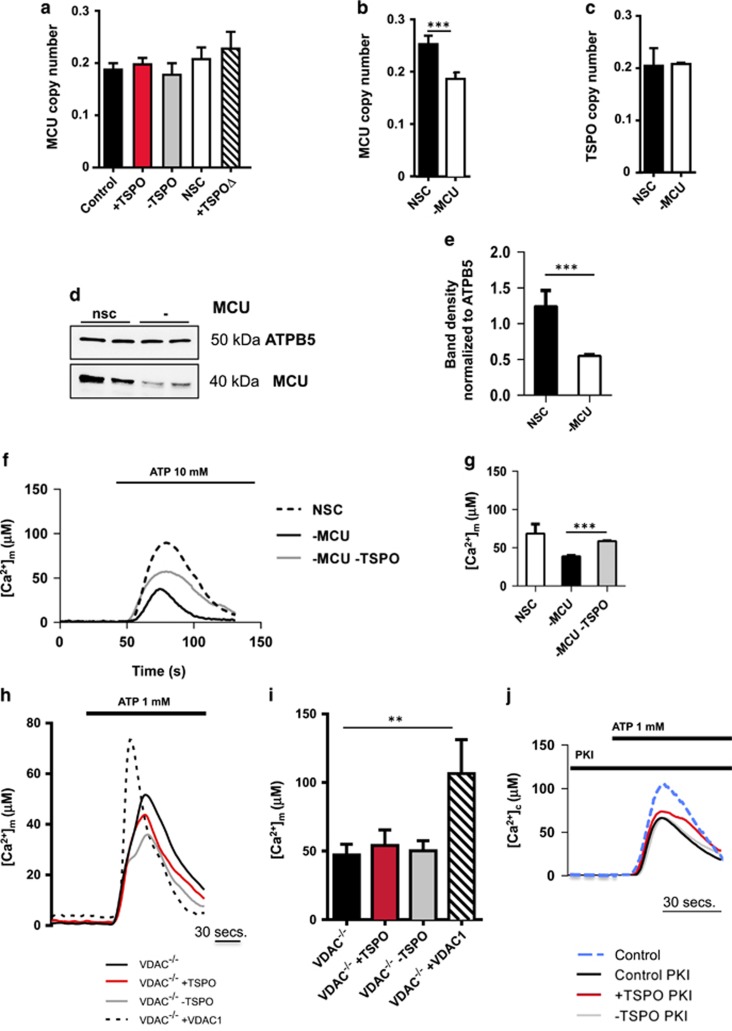
TSPO-dependent Ca^2+^ regulation occurs at the OMM and requires VDAC1 and PKA. (**a**) Real-time qRT-PCR studies show mRNA levels of MCU, normalized to levels of VDAC in MEFs; *n*=3; *P*>0.05. (**b**) Real-time qRT-PCR studies show mRNA levels of MCU, normalized to levels of VDAC in MEFs with modulated MCU expression; *n*=7; ***P<0.001. (**c**) Real-time qRT-PCR studies show mRNA levels of TSPO, normalized to levels of VDAC in MEFs with modulated MCU expression, *n*=5. (**d** and **e**) Immunoblot of MCU in MEFs WT and knocked out for the gene with quantification in **e**. (**f**) Representative traces showing ATP-induced mitochondrial Ca^2+^ uptake in MEFs. (**g**) Graph showing the mean maximum [Ca^2+^]_m_ in response to ATP (1 mM) in MEFs (*n*=3; ***P<0.001). (**h**) Representative traces showing ATP-induced Ca^2+^ transients in TSPO-silenced VDAC1^−/−^ MEFs. (**i**) Bar chart showing the mean maximum [Ca^2+^]_m_ in VDAC1^−/−^ MEFs; *n*=4; **P<0.05. (**j**) Representative traces showing ATP-induced Ca^2+^ transients MEFs exposed to PKI

The uptake of Ca^2+^ across the inner mitochondrial membrane (IMM) is dependent on the mitochondrial Ca^2+^ uniporter (MCU).^42^ To understand whether TSPO could be acting at this level, we verified the impact of TSPO expression modulation on the MCU transcription. Quantitative real-time RT-PCR was performed^42^ on samples extracted from TSPO-silenced MEFs with no differences recorded (Control: 0.19±0.01, +TSPO: 0.20±0.01, −TSPO: 0.18±0.02, non-silencing control (NSC): 0.21±0.02, +TSPO^Δ150–158^: 0.23±0.03; Figure 4a).

We subsequently knocked down MCU in MEFs (qRT-PCR NSC: 0.26±0.02, −MCU: 0.19±0.02, Figure 4b, western blotting analyses in Figures 4d and e) and checked whether this had effect on TSPO expression *per se*, which did not (NSC: 0.21±0.04, −MCU: 0.22±0.03, Figure 4c).

Mitochondrial Ca^2+^ uptake was then assessed in MCU/TSPO-silenced MEFs by using the mAeq.^31^ As expected, knocking down of MCU resulted in a significant decrease of the mitochondrial Ca^2+^ capacity (38.81±1.2 *μ*M) compared with NSC (89.78±12.2 *μ*M; Figures 4f and g). Interestingly, when TSPO expression was also suppressed, mitochondrial Ca^2+^ uptake was partially rescued (58.68±1.2 *μ*M; Figures 4f and g). By reducing TSPO expression and therefore increasing the amount of Ca^2+^ within the intermembrane space, the lower levels of MCU might still be active and therefore permit influx of Ca^2+^ into the matrix, leading to a partial recovery of the mitochondrial Ca^2+^ uptake capacity.

As TSPO interacts with VDAC1, which is the principal Ca^2+^ channel of the OMM,^43, 44^ we measured ATP-induced mitochondrial Ca^2+^ uptake in TSPO-silenced VDAC1 knockout MEFs (VDAC1^−/−^ MEFs^45^). In these cells, modulation of TSPO failed to alter the ATP-induced [Ca^2+^]_m_ signal thus indicating that VDAC1 is essential for its effects on [Ca^2+^]_m_ (representative traces reported in Figure 4h and quantified data in **I**, data are *μ*M Ca^2+^, VDAC1^−/−^: 47.09±7.9; VDAC1^−/−^ +TSPO: 54.09±11.4; VDAC1^−/−^ −TSPO: 50.15±7.4; VDAC1^−/−^ +VDAC1: 106.6±24.8; *n*>4; *P*<0.05). Still, the actual regulatory pattern remained elusive. Though, it was previously demonstrated that VDAC1 can be phosphorylated by the PKA^46^ leading to reduction in VDAC1 conductance.^47^ Equally, it was also shown that TSPO forms a macromolecular complex with the ACBD3, PKA and VDAC1 on the OMM.^48^ To pharmacologically test whether PKA contributes the impairment of the mitochondrial Ca^2+^ signaling recorded when TSPO is overexpressed, we treated MEFs with a synthetic protein kinase inhibitor peptide (PKI) and re-assessed mitochondrial Ca^2+^ levels in control and TSPO knockdown cells.^49^

The PKA inhibitor (used as 1 mg/ml), which binds the free catalytic subunit of PKA and prevents phosphorylation of the kinase targets,^49^ normalized the differences in mitochondrial Ca^2+^ transients (Figure 4j) indicative of active role by the VDAC phosphorylation.

### TSPO regulation of ACBD3/PKA mitochondrial recruitment and VDAC1 phosphorylation

**Figure 5:**
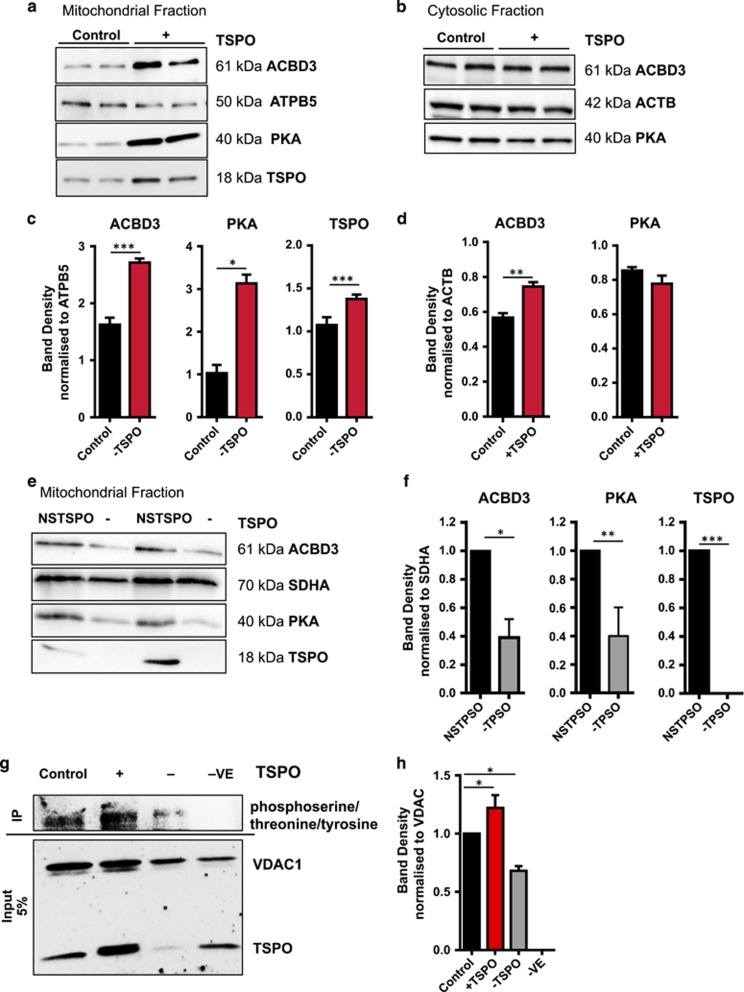
TSPO recruits ACBD3 and PKA to mitochondria and regulates VDAC1 phosphorylation. (**a**) Immunoblot of mitochondrial and (**b**) cytosolic extracts from control and +TSPO MEFs run in duplicate with antibodies indicated on the right. (**c**) Bar charts showing the mean band density of ACBD3, PKA, TSPO normalized to ATPB5 in mitochondrial fractions (*n*=4) and (**d**) ACBD3, PKA normalized to ACTB in cytosolic fractions. (**e**) Immunoblot analysis of mitochondrial ACBD3 and PKA in SH-TSPOkd. (**f**) ACBD3, PKA, TSPO normalized to SDHA in mitochondrial fractions (*n*=4). (**g**) Immunoprecipitation of VDAC from SH-SY5Y cells in TSPO-modulated conditions. The lower section (input 5%) western blotted with VDAC1 and TSPO antibodies. The upper section of the blot (IP) shows phosphorylated VDAC1. (**h**) Bar chart showing the mean band density of *α*-phosphoserine/threonine/tyrosine according to TSPO expression, normalized to input VDAC1 (*n*=3; *P<0.05, **P<0.01, ***P<0.001)

In light of the above results, we examined the mitochondrial recruitment of both the ACBD3 and PKA. In mitochondrial fractions isolated from TSPO-overexpressing cells, we observed an increase of both ACBD3 and PKA, as shown by immunoblotting experiments reported in Figure 5a with the corresponding band density analysis in Figure 5c (ACBD3, Control: 1.63±0.19; +TSPO: 2.72±0.07; PKA, Control: 1.08±0.09; +TSPO:3.13±0.20; TSPO, Control: 1.03±0.19; +TSPO: 1.38±0.05). The analysis of cytosolic fractions is shown in Figure 5b with band density analysis in Figure 5d (ACBD3, Control: 0.57±0.03; +TSPO: 0.75±0.02; PKA, Control: 0.85±0.02; +TSPO: 0.78±0.05). The same analysis was repeated in mitochondrial fractions of SH-TSPOkd cells (Figures 5e and f) that showed a significant decrease in accumulation of both ACBD3 (Control: 1.00±0.00; −TSPO: 0.39±0.13) and PKA (Control: 1.00±0.00; −TSPO: 0.40±0.20), compared with WT SH-SY5Y (Figure 5f).

VDAC1 phosphorylation was tested by probing the co-immunoprecipitated TSPO and VDAC1 with anti-phosphoserine/threonine/tyrosine (Figure 5g) showing that VDAC1 was indeed phosphorylated more when TSPO is overexpressed (+TSPO, 1.22±0.11) and significantly less in −TSPO conditions (−TSPO, 0.68±0.04) compared with Control (Figure 5h). Further evidence for specific PKA involvement in this process of post-translational modification of VDAC1 was obtained by comparing the PKA translocation in WT and VDAC1^−/−^ cells (Supplementary Figure 4e). In WT cells under oxidative stress, PKA readily accumulates on mitochondria, whereas in VDAC^−/−^ cells this is abolished. In the same conditions, the mitochondrial portion of PKCε remains unaltered indicative of a PKA-specific event (Supplementary Figure 4f).

### Glutamate induces TSPO expression and accumulation of ROS paralleled by mitochondrial recruitment of the ACBD3/PKA complex

**Figure 6:**
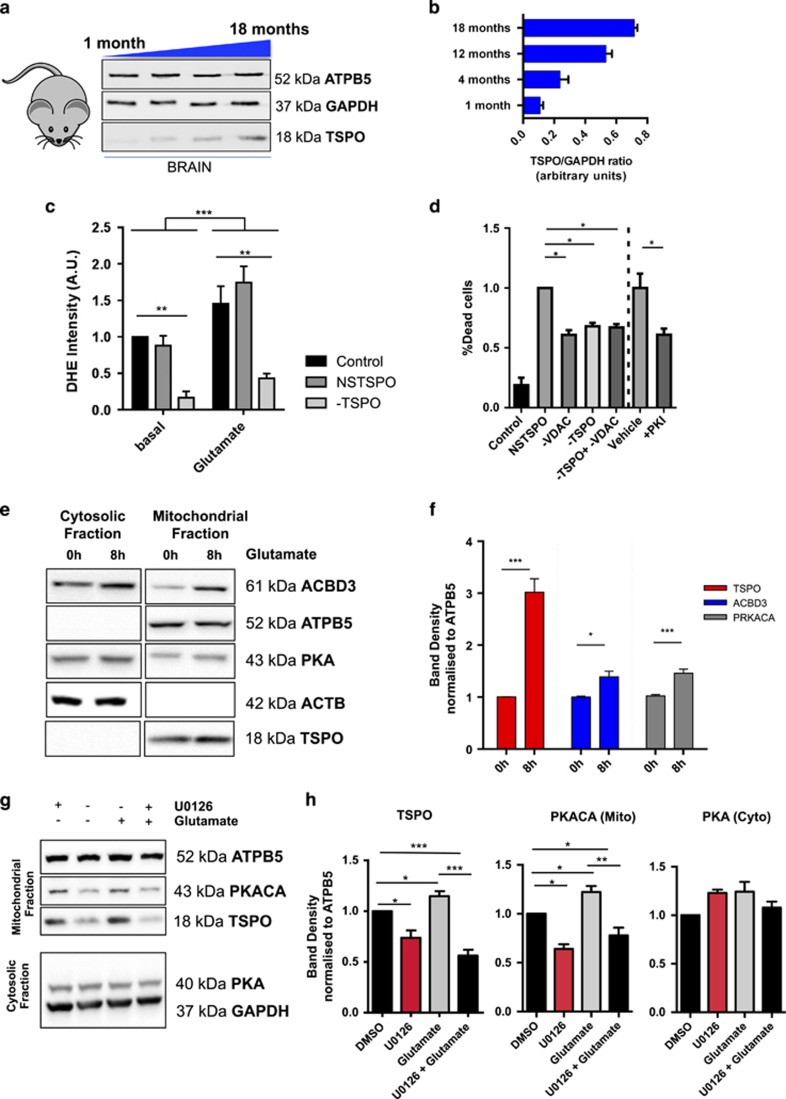
Glutamate-induced stress response upregulates TSPO recruiting the ACBD3–PKA complex on mitochondria. (**a**) TSPO expression level in brain lysates isolated from mice at different time points −1, 4, 12 and 18 months. (**b**) Data quantification (*n*=2). (**c**) Bar chart showing the rate of increase in DHE fluorescence intensity in control, non-silencing control and TSPO-modulated cells treated with glutamate (4 mM, acute; ⩾15 cells per field of analysis, *P*<0.01). (**d**) Bar chart showing cell death induced by glutamate in VDAC- or TSPO-modulated cells, or cells treated with PKI (*n*=4, *P*<0.05). (**e**) Representative immunoblot of mitochondrial and cytosolic fractions isolated from SH-SY5Y treated with glutamate. Antibodies are indicated on the right. (**f**) Bar chart showing band density changes in mitochondrial distribution of TSPO, ACBD3 and PKA over the duration of glutamate treatment, normalized to ATPB5. (**g**) Representative immunoblot of mitochondrial and cytosolic fractions of neurons challenged with glutamate during co-treatment with U0126. (**h**) Bar charts reporting the relative quantification of the proteins expression profile (*P<0.05, **P<0.01, ***P<0.001)

*In vivo* imaging analysis had previously shown that TSPO accumulates in aged states of the brain;^6^ here, by sampling brain protein extracts from wild-type mice at 18 months, we reconfirmed the positive correlation between ageing and TSPO (1 month: 0.11±0.02; 4 months: 0.23±0.05; 12 months: 0.53±0.04; 18 months: 0.72±0.02; Figure 6b).

The aged brain is highly sensitive to glutamate-induced toxicity and we therefore explored the TSPO-coordinated molecular cascade in SH-SY5Y cells challenged with glutamate to provide *in vitro* evidences of a pathophysiological stress condition known to exert cytotoxicity exploiting both Ca^2+^ and ROS.^50^ Following glutamate application (4 mM, acute), an increase in ROS production was immediately detected (Figure 6c, Supplementary Figure 4a), abolished in −TSPO cells (WT basal: 1, NSC basal: 0.88±0.14, −TSPO: 0.17±0.08, WT+glutamate: 1.45±0.24, NSC+glutamate: 1.69±0.22, −TSPO+glutamate: 0.43±0.07, *n*=5). Correspondingly, in these cells, glutamate-induced cell death (20 mM, 8 h) was far lower (Figure 6d). In keeping with this, VDAC suppression as well as PKI treatment in TSPO expressing cells did mediate the same protective outcome (untreated: 0.19±0.06, NSC: 1±0.09, −VDAC: 0.61±0.04, −TSPO 0.68±0.03, −TSPO±VDAC: 0.67±0.03, vehicle: 1±0.00, +PKI: 0.61±0.05, *n*=3).

The detrimental effect of glutamate in WT SH-SY5Y was paralleled by TSPO, ACBD3 and PKA accumulation in mitochondrial fractions (Figure 6e) suggesting a pathophysiological adaptation following stress-induced TSPO expression (fold increase as seen in Figure 6f, TSPO: 3.2±0.35, ACBD3: 1.2±0.021, PKA: 1.18±0.018).

As a further control, we tested MEFs stably knocked out for PKCε (PKCε^−/−^) in which TSPO expression is largely impaired.^51^ In these cells, glutamate led to negligible TSPO upregulation and abolished mitochondrial accumulation of ACBD3 and TSPO (Supplementary Figures 4b–d).

**Figure 7:**
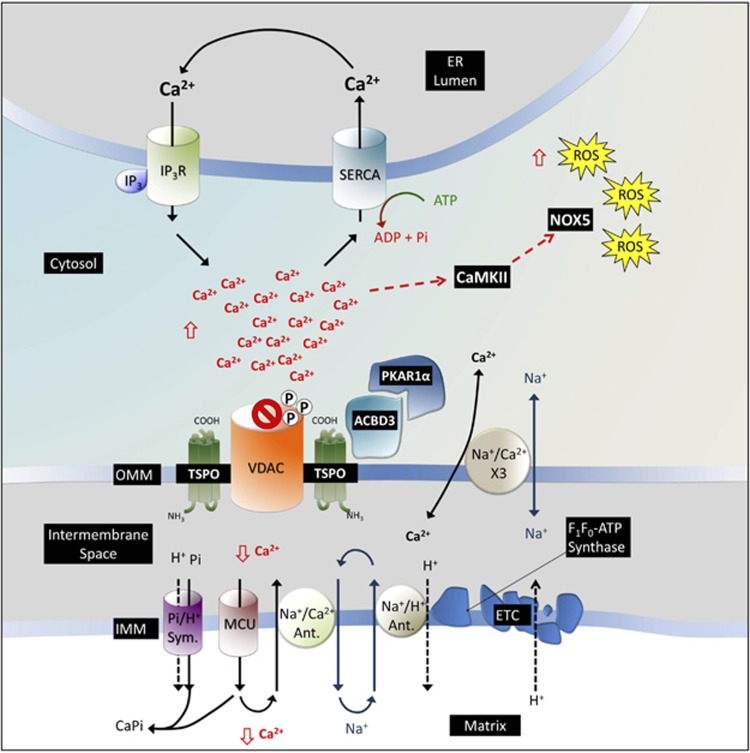
Working mechanism for TSPO-mediated regulation of mitochondrial Ca^2+^ handling. Overexpression of TSPO results in the recruitment of ACBD3 and PKA and corresponds with an increase in VDAC1 phosphorylation. Ca^2+^ released from internal stores accumulates in the cytosol, unable to diffuse across the outer mitochondrial membrane through VDAC1. The increase in cytosolic Ca^2+^ activates NOX5, via CaMKII leading to altered REDOX balance

Finally, to corroborate the pathway further, we pursued a pharmacological approach using U0126 (10 *μ*M × 48 h), a highly selective inhibitor of the extracellular signal-regulated kinase (ERK) to prevent stress-induced transcription of TSPO^51^ (Figure 6g). U0126 prevents the mitochondrial accumulation of TSPO when co-administrated with glutamate on neuronal cells (Figures 6g and h; glutamate+UO126, TSPO: 0.56±0.09, PRKACA: 0.79±0.11). Similarly, the mitochondrial accumulation of PKA is impaired too implying an overall inhibition of the pathway (Figures 6g and h). Hence, we generated the working model proposed in Figure 7.

## Discussion

Mitochondrial Ca^2+^ homeostasis is crucial for balancing cell survival and death.^52^ The regulatory mechanisms of mitochondrial Ca^2+^ uptake across the IMM are especially important for the regulation of ATP synthesis, mitochondrial fission–fusion dynamics and the generation of reactive oxygen species.^53, 54^ Mitochondrial Ca^2+^ influx is regulated by a single transport mechanism, the mitochondrial Ca^2+^ uniporter (MCU);^42^ although additional elements can influence the kinetics and efficiency of mitochondrial Ca^2+^ uptake.^39, 44, 55^ Among these, the voltage-dependent anion-selective channels, which shuttle Ca^2+^ and metabolites across the OMM, are of immediate interest given the interaction with TSPO^3, 17^ and the latter being an indicator of cell and tissue pathology^6^ recently linked to defective mitochondrial autophagy.^17^

Here we demonstrate that mitochondrial TSPO reduces the transfer efficiency of Ca^2+^ into the organelle via VDAC1 and this is observed both in human, murine and canine cells, suggesting a conserved mechanism for mitochondrial Ca^2+^ adaptation. Furthermore, having recorded identical signaling pattern in microglial cells (Supplementary Figures 2f–h), the activation of which underlies brain conditions imaged by TSPO,^56^ implies a likely outcome on the pro-inflammatory intracellular pathways (e.g., Nf-*κβ*) mastered by the Ca^2+^ and ROS signaling homeostasis.^57^

TSPO overexpression deregulates mitochondrial Ca^2+^ uptake capacity and increases the maximum [Ca^2+^]_c_ following challenge with IP_3_ generating stimuli (Figures 2a–d,Supplementary Figures 1b–e and 2a–d), and pharmacological inhibition of Ca^2+^ retention by intracellular stores (Figures 2e–h). This is consistent with previous evidences obtained with the endogenous TSPO ligand, hemin (iron protoporphyrin IX), which limits mitochondrial Ca^2+^ accumulation in isolated mitochondria by reducing VDAC conductance.^16^ Synthetic TSPO ligands can also affect mitochondrial Ca^2+^ handling.^15^

TSPO leaves unaltered communication and connectivity between the ER and mitochondria (Figures 3d–g) consistently with previous data on mitofusin 1 and 2 levels that also remain constant.^17^

The TSPO-mediated inhibition of mitochondrial Ca^2+^ uptake occurs independently from the MCU too. The observed null effect (Figure 4a), though, does not rule out that TSPO could facilitate the activity of MCU by alimenting surrounding Ca^2+^ pools as knocking down both TSPO and MCU partially restored the [Ca^2+^]_m_ (Figures 4f and g).

The TSPO-mediated regulation of mitochondrial Ca^2+^ transients appears nonetheless depending on VDAC1 (Figures 4h and i) which is, in TSPO-dependent manner, phosphorylated by the PKA recruited to the mitochondrion in complex with ACBD3 (Figures 5a–f). VDAC1 phosphorylation results in diminishing its conductance for Ca^2+^ (Figures 5g and h).^46, 58^

Interestingly, high PKA activity is reported to promote mitochondrial elongation,^58^ by phosphorylating DRP1 at serine 656,^59^ which is in apparent contrast with our observations on mitochondrial structure (Figure 5a). It must, nonetheless, be noted that TSPO also promotes a rise in [Ca^2+^]_c_, which could activate Ca^2+^ kinases (Cdk1/cylin B or CaMK)^59, 60, 61^ thus phosphorylating DRP1 at alternative sites (e.g., ser-585 and ser-616) antagonizing, and not compensating, for PKA-induced inhibitory phosphorylation at ser-656.

Non-physiological rises in [Ca^2+^]_c_ can nonetheless predispose the cells to oxidative stress via activation of NOXs. Experiments performed using the pharmacological NOX enzyme inhibitors DPI, Apocynin, as well as KN93 that instead targets CaMKII (Figures 1d–f), suggest this to be the most likely signaling pathway via which TSPO triggers intracellular oxidative stress in mammalian cells. This was confirmed by gene expression analysis in which a correlation between TSPO expression and the Ca^2+^-regulated isoform of NOX was observed (Figures 1j–l). As this isoform of the NOX family is implicated in various pathological conditions as well as the regulation of redox-sensitive signaling pathways upstream of TSPO, such as ERK1/2, it could well represent the pivotal means for the feed-forward mechanism – hypothesized in precedence^17^ – whereby the expression of TSPO increases during redox stress and to which it directly contributes. This Ca^2+^-regulated mechanism of ROS accumulation could explain the TSPO-mediated inhibition of oxidative stress-sensitive processes such as the prevention of PARK2-mediated mitophagy.^17^

The increased rate of cell death recorded *in vitro* in TSPO-overexpressing cells exposed to glutamate, accounts for greater susceptibility to high dosage of the neurotransmitter (Figure 6), and given the progressive, age-dependent accumulation of TSPO in the brain (Figures 6a and b), it would be worth exploring whether this could act as a predictive molecular signature to neurodegeneration. The mitochondrial accumulation of ACBD3 levels, which parallel those of TSPO (Figures 6e and f), has been previously linked to NDMA neurotoxicity.^62, 63^

Equally, even though the recruitment of cytosolic elements on mitochondria has been previously reported,^64^ (Figure 6c) never before was this ascribed to master mitochondrial Ca^2+^ uptake.

An untested scenario is whether the overexpression of TSPO consequent acute intoxication (e.g., high glutamate level), or similar forms of toxicological stress, may prime alternative ways of cell death in which the imbalance of redox homeostasis has a great part. It is indeed intriguing to think that TSPO as a priming element of the oxytosis, of the above-mentioned typologies of cell death, is the prototype.^65, 66^ We indeed reported that the sole TSPO overexpression depletes intracellular GSH level in MEFs,^17^ thus implying that protein could be a molecular conduit in this very process of cell execution, and future studies shall clarify on this.

Neuronal demise induced by the overstimulation of glutamate receptors is dependent on a sustained increase in [Ca^2+^]_i_^67^ in which mitochondria are implicated as buffering compartments capable of modulating the degree of cellular Ca^2+^ tonicity.^68^ Molecules that regulate mitochondrial Ca^2+^ dynamics may indeed prove fundamental in reducing the severity of cellular insults to prevent or delay onset of neurodegeneration^69^ as well as regulating other processes, such as Autophagy, which are influenced by the Ca^2+^ tonicity.^70^

TSPO may therefore represent part of an innate stress response pathway to prevent accumulation of Ca^2+^ in the mitochondria during cytotoxic insults. However, in turn, this may lead to the accumulation of both short- and long-lived redox molecules, which would underline detrimental effects for which the overexpression of TSPO could represent both a diagnostic and therapeutic target.^71^

## Materials and methods

### Cell culture and transfection

Mouse embryonic fibroblast cells (MEFs)^17, 45^— a kind gift from Professor William Craigen (Bayer College of Medicine Medical Genetics Laboratories, Houston, TX, USA) and canine mammary gland epithelial cells (CF35), SH-SY5Y neuroblastoma (ATCC, Manassas, VA, USA) were maintained in a temperature-controlled, humidified incubator at 37 °C with 5% CO_2_ (Hera Cell 240, Thermo Scientific, Essex, UK) in Dulbecco’s modified Eagle medium (DMEM; Life Technologies, 11995065) supplemented with 10% fetal bovine serum (FBS; Life Technologies, 10082147), 100 U/ml penicillin and 100 mg/ml streptomycin (Life Technologies, 15140122).

TSPO was depleted using siRNA before experimentation as described previously.^17^ To knock down MCU in MEFs, we used siRNA 5′-GCCAGAGACAGACAATACT-3′.^42^ As a control for these studies, a non-silencing siRNA sequence (AllStars Negative Control, Qiagen, 1027281) was transfected. For overexpression studies, a pcDNA3.1 (−) construct containing the full murine TSPO cDNA open reading frame, was transfected; the empty vector was used as a control in this instance. The cells were transfected using a standard Ca^2+^ phosphate method. All experiments with modulation of TSPO expression were performed 48 h post transfection.

### Quantitative real-time PCR

One microgram total RNA was reverse transcribed using iScript select cDNA synthesis kit (Bio-Rad). Nox/Duox enzyme expression was determined via quantitative real-time PCR (qPCR) using gene-specific Taqman gene expression assays (Applied Biosystems, Foster City, CA, USA). Each reaction was performed in a final volume of 11 *μ*l containing 50 ng cDNA, 0.55 *μ*l 20 × gene-specific Taqman assay and 5.5 *μ*l 2 × ABI FAST Mastermix (Applied Biosystems). Expression was normalized to the endogenous reference TATA-Box-binding protein (TBP) (forward 5′-CACGAACCACGGCACTGATT-3′ reverse 5′-TTTTCTGCTGCCAGTCTGGAC-3′ probe 5′-FAM-TCTTCACTCTTGGCTCCTGTGCACA-TAMRA-3′). For TBP, the primers were added to a final concentration of 900 nM, and the final probe concentration was 150 nM. Real-time PCR conditions were as follows: one cycle of hold at 50 °C for 2 min and one cycle of polymerase activation at 95 °C for 20 s, followed by 40 cycles of 95 °C for 3 s and 60 °C for 30 s on an ABI Prism 7500 Fast RT-PCR System (Applied Biosystems). Amplifications were performed in duplicate with the exception of Nox5, which was performed in quadruplicate due to its relative low abundance. Fold change in gene expression was calculated according to the 2^−ΔΔCT^ method (Livak and Schmittgen 2001 PMID: 11846609) from four independent experiments.

### NOX5 lentiviral knockdown

Lentiviral particles for scrambled control and two different Nox5 small hairpin RNA (shRNA) sequences were generated in HEK293FT cells (Invitrogen, Carlsbad, CA, USA) using the packaging plasmids pMD2.G and psPAX2 (Addgene; Cambridge, MA, USA) and the lentiviral vector pLKO.1 containing Nox5-specific shRNA (clone ID TRCN0000046099 or TRCN0000046101 from Dharmacon, GE Lifesciences, USA) or scrambled control (Addgene plasmid 1864). Lentiviral titer was determined via application of serially diluted lentivirus-containing supernatant to U2-OS cells. After 24 h, puromycin selection was initiated (0.5 *μ*g/ml), and 6 days later, colonies counted following staining with 1% crystal violet (Sigma-Aldrich, St. Louis, MO, USA). For lentiviral infection, 100 000 SH-SY5Y cells were seeded in 25 cm^2^ flasks. The next day, culture medium containing 8 *μ*g/ml hexadimethrine bromide (Sigma-Aldrich) and lentiviral particles at a multiplicity of infection of 40 was added to the cells. The infection was repeated again 48 h later. Puromycin selection (0.5 *μ*g/ml) was started 48 h after the second infection, and the concentration doubled at 48 h intervals until the final selection concentration (2 *μ*g/ml) was reached. Three independent experiments were performed generating three independent polyclonal sublines for each shRNA.

### TSPO knockdown in SH-SY5Y

This was achieved using the pGIPZ shRNA vector: clone ID V3LHS_331646, target sequence: 5′-TGAGTGTGGTCGTGAAGGC-3′, purchased from Open Biosystems (Huntsville, AL, USA).

Non-silencing shRNA vector sequences (mature antisense): V3LHS_331648: 5′-ACGCAGTAGTTGAGTGTGG-3′ V3LHS_331650: 5′-TCTGCAGGCCGGCGTACCA-3′.

Transfection was performed using standard Ca^2+^ phosphate-based protocol,^72^ and the cells maintained for 2 weeks in media supplemented with 3 *μ*g/ml puromycin (SERVA Electrophoresis GmbH) for the selection of transfected (GFP-positive) cells. TSPO downregulation in SH-TSPOkd cells was confirmed by western blot analysis: SH-SY5Y and SH-TSPOkd cells were washed in PBS and collected by gentle scraping in ice-cold RIPA buffer. Protein concentration was estimated using the Bradford reagent. Following this, 50 *μ*g of protein was mixed with Laemmli sample buffer and boiled at 95 °C for 2 min. The samples were resolved by sodium-dodecyl sulfate polyacrylamide gel electrophoresis (SDS-PAGE) and transferred to nitrocellulose membranes. The blots were probed with antibodies diluted in 1% bovine serum albumin in tris-buffered saline with 0.1% Tween-20 (TBS-T) overnight (4 °C). Then, they were detected using horseradish peroxidase-conjugated secondary antibody (1 : 2000; Cell Signaling, Danvers, MA, USA; 60 min at room temperature (RT) in 5% non-fat milk) and an enhanced luminescence kit (Amersham Pharmacia Biotech, Piscataway, NJ, USA). The antibodies used were rabbit monoclonal anti-TSPO (Abcam, UK; ab109497), mouse monoclonal anti-GAPDH (Abcam ab9482), goat anti-rabbit IgG and goat anti-mouse IgG horseradish peroxidase-conjugated secondary antibodies (Bio-Rad).

### Immunoblotting analyses

For whole-cell lysates, the cells were collected and resuspended in 1 ml of lysis buffer, 50 mM Tris (Sigma T6066) pH 8.0; 150 mM NaCl; 1% Triton-X (Sigma, T9284), protease inhibitor (Roche, UK, 04693132001) and left on ice for 20 min. The cell debris was removed by centrifugation at 17 000 G for 20 min. For mitochondrial isolation, 500 *μ*l sucrose isotonic fractionation buffer (250 mM sucrose; 20 mM HEPES (pH 7.4); 10 mM KCl; 1.5 mM MgCl_2_ (M4880); 1 mM EDTA (Sigma, E6758); 1 mM EGTA (Sigma, E3889); protease inhibitor) was added to confluent cells growing on 10 cm plates. The plates were scraped immediately and the cell suspension was then passed through a 26-gauge needle 12 times before centrifuging at 700 × *g* for 5 min. The resulting supernatant was subjected to a further centrifugation at 10 000 × *g* to obtain the mitochondrial pellet. The proteins were resolved on 12% SDS-PAGE gels, transferred onto nitrocellulose membranes and detected with *α*-TSPO (Abcam ab109497), ATPB5 (Abcam ab14730), ACBD3 (Abcam ab131592), ACTB (Abcam ab8226), PRKACA (Abcam ab76238), Cyt-C (Abcam ab110325) *α*-GAPDH (Abcam ab9482).

### Immunoprecipitation studies

TSPO expression in wild-type SH-SY5Y cells was modulated by transfection with TSPO cDNA or anti-TSPO siRNA. The cells were washed once in PBS then lysed by suspension in lysis buffer (20 mM Tris, 100 mM NaCl, pH 7.5) at 4 °C for 30 min. The unbroken cells and nuclei were removed by centrifugation at 2600 × *g* for 5 min. The supernatants were immunoprecipitated using a dynabead co-immunoprecipitation kit (Invitrogen, 14321D) following the manufacturer’s instructions. Briefly, for each reaction, 5 *μ*g of *α*-VDAC antibody was coupled to 1 mg of dynabeads using the provided coupling solutions overnight at 37 °C. For negative control conditions, the beads were subjected to the same procedure in the absence of VDAC antibody. The supernatants were mixed with the antibody-coupled beads and incubated for 1 h at 4 °C with end-over-end mixing. Boiling the washed beads in SDS-PAGE loading buffer eluted complexes. The proteins were resolved on 12% SDS-PAGE gels, transferred onto nitrocellulose membranes and detected with *α*-Phosphoserine/threonine/tyrosine antibody (Abcam 15556), *α*-VDAC (Abcam ab14743) *α*-TSPO (Abcam ab109497) and *α*-GAPDH (Abcam ab9482) antibodies.

### Immunocytochemistry

Primary co-cultures of neurons and astrocytes were fixed in 4% PFA (15 min, RT) and permeabilized in 0.5% Triton-X (15 min, RT). Blocking was carried out for 1 h at RT in 10% Goat Serum (Life Technologies, 16210064) and 3% BSA in PBS. Primary antibody incubations were conducted overnight for 16 h at 4 °C in blocking solution in a humidified chamber. After a further wash step, secondary antibodies were incubated for 1 h in blocking solution at RT, before a final wash step. Finally, the cells were mounted on slides with 4′,6-diamidino-2-phenylindole (DAPI) mounting medium (Abcam, ab104139) and sealed.

### Ca^2+^ recordings

The mtAEQmut transfected cells grown on glass coverslips were incubated with 5 *μ*M coelenterazine (Promega, Madison, WI, USA, S2001) for 2 h in recording medium (RM) supplemented with 1 mM CaCl_2_.^17^ Glass coverslips were then transferred to the thermostatted perfusion chamber of a custom-built luminometer (Cairn Research, Faversham, Kent, UK). Agonists and other drugs, as indicated in the figure legends, were added to the same RM. The experiments were terminated by lysing cells with 100 *μ*M digitonin in a hypotonic Ca^2+^-rich solution (10 mM CaCl_2_ in H_2_O), so as to discharge the remaining aequorin pool. The aequorin luminescence was captured by a photon-counting board and the data were calibrated into [Ca^2+^] values using a computer algorithm.^73^

Alternatively, TSPO knockdown cells on glass coverslips were loaded with 8 *μ*M rhod-2 AM (Life Technologies, Paisley, UK) and 0.005% pluronic in modified Kreb’s Ringer Buffer (mKRB; 125 mM NaCl; 5 mM KCl; 1 mM MgSO_4_; 1 mM Na_2_HPO_4_; 5.5 mM glucose; 20 mM NaHCO_3_; 2 mM l-glutamine; 20 mM HEPES supplemented with 1.8 mM CaCl_2_; pH 7.35) for 35 min at 37 °C.^74^ Following loading, the cells were washed four times in mKRB before imaging. For cytosolic Ca^2+^, the cells were loaded with 5 *μ*M Fluo-4 AM (Life Technologies, Paisley, UK) and 0.02% pluronic in mKRB for 30 min at 37 °C. The cells were immediately transferred to a Leica SP5 Confocal microscope for imaging where they were maintained at 37 °C. A field of transfected cells was selected (either YFP positive for mitochondrial Ca^2+^ or mtRFP positive for cytosolic Ca^2+^) and using a × 40 objective, a time series was then set up so that a new image was acquired every 1 s to ensure the transient Ca^2+^ increase is captured. For mitochondrial Ca^2+^ accumulation, the 543 nm laser was used to excite Rhod-2 (red) and for cytosolic Ca^2+^, the 488 nm laser was used to excite Fluo-4 (green). After continuous recording for approximately 30 s, ATP was added (100 *μ*M–1 mM). The increase in Ca^2+^ concentration induces a sharp rise in fluorescence intensity of both Fluo-4 and Rhod-2.^74^ When the signal returned to near starting values, ionomycin (Sigma, I3909) was added (0.5 *μ*M). The application of ionomycin eliminates any remaining Ca^2+^ gradient between all membranes, thus saturating available Fluo-4/Rhod-2. Recording was stopped when the intensity of the Ca^2+^ dye reached a plateau. The data were normalized to the ionomycin response by using the equation (*x*−min *x*)/(max *x*−min *x*).

### Reactive oxygen species recording

To quantify cellular levels of ROS^17^ in this study, the commonly used fluorescent dye, dihydroethidium (DHE), was used. DHE is sensitive to O_2_^−^ (superoxide) and may be oxidized. Once oxidized, DHE binds to DNA, which results in the amplification of the red signal within the nucleus. The rate at which the signal increases in intensity is dependent on cytosolic levels of O_2_^−^, thereby enabling the quantification of ROS levels within the cell. The cells grown on glass coverslips were transfected with mitochondria-targeted GFP (mtGFP) and TSPO expression silenced. A field of healthy transfected cells was selected and 5 *μ*M DHE (Life Technologies, D11347), diluted in RM, was added. The rate of increase in fluorescence intensity was measured through continuous recording for at least 10 min.

### Mitochondrial membrane potential (ΔΨ_m_) analysis

The cells, co-transfected with YFP were loaded with 50 nM tetramethyl rhodamine methyl ester (TMRM) (Sigma, T5428) in HEPES-buffered salt solution (156 mM NaCl; 3 mM KCl; 2 mM MgSO_4_; 2 mM CaCl_2_; 10 mM glucose; 10 mM HEPES). The cells were allowed to equilibrate the dye for at least 30 min at room temperature before they were transferred to a Zeiss LSM 510 confocal microscope (× 40 objective) for imaging. Mitochondrial ROIs were demarcated and the corresponding TMRM fluorescence intensities calculated by removing background signal through thresholding as previously explained.^72, 74^

### Mitochondrial morphology analysis

The CF35 cells were transfected with mtGFP to visualize mitochondria. To assess mitochondrial morphology, the mitochondrial form factor (FF) and aspect ratio (AR) were calculated as described previously.^74^ For measuring ER-mitochondria connectivity, MEFs transfected with mtRFP were loaded with ER tracker green (Life Technologies, E34251; 1 *μ*M) for 30 min. An object-based estimate of the co-localization events between ER-green and mtRFP channel 3D z-stacks was obtained by calculating Manders’ coefficients (M1 and M2).^74^ These are based on the Pearson’s correlation coefficient but do not consider average intensity values and vary from 0 (non-overlapping) to 1 (100% co-localization).

### Preparation of brain tissue protein lysates

The 1-, 4-, 12- and 18-month-old C57/BL6 mice were killed by cervical dislocation and brains harvested on ice. The frontal cortex tissue was excised by forceps and lysed in a specific lysis buffer containing 1% Triton X-100 (Applichem Panreac), 320 mM sucrose (Sigma-Aldrich), 50 mM Tris-HCL pH 7.5, 50 mM NaCl (Sigma-Aldrich), supplemented with protease inhibitor cocktail solution (Sigma-Aldrich). Tissue was disrupted by sonication, followed by 30 min incubation on ice to allow protein solubilization. Next, the lysates were cleared by centrifugation for 20 min at 13 000 r.p.m. at 4 °C. The supernatants were then collected and protein concentration measured using Bradford method (Applichem Panreac, Germany). The protein samples were stored at −80 °C before western blot analysis.
