# Supplementary material for: A role for TSPO in mitochondrial Ca2+ homeostasis and redox stress signaling
Source: Cell Death Dis. 2017 Jun 22;8(6):e2896–. doi: 10.1038/cddis.2017.186 (PMC5520880; doi:10.1038/cddis.2017.186)
Supplement: Supplementary Figures [file cddis2017186x1.pdf]

Suppl. Fig. 1

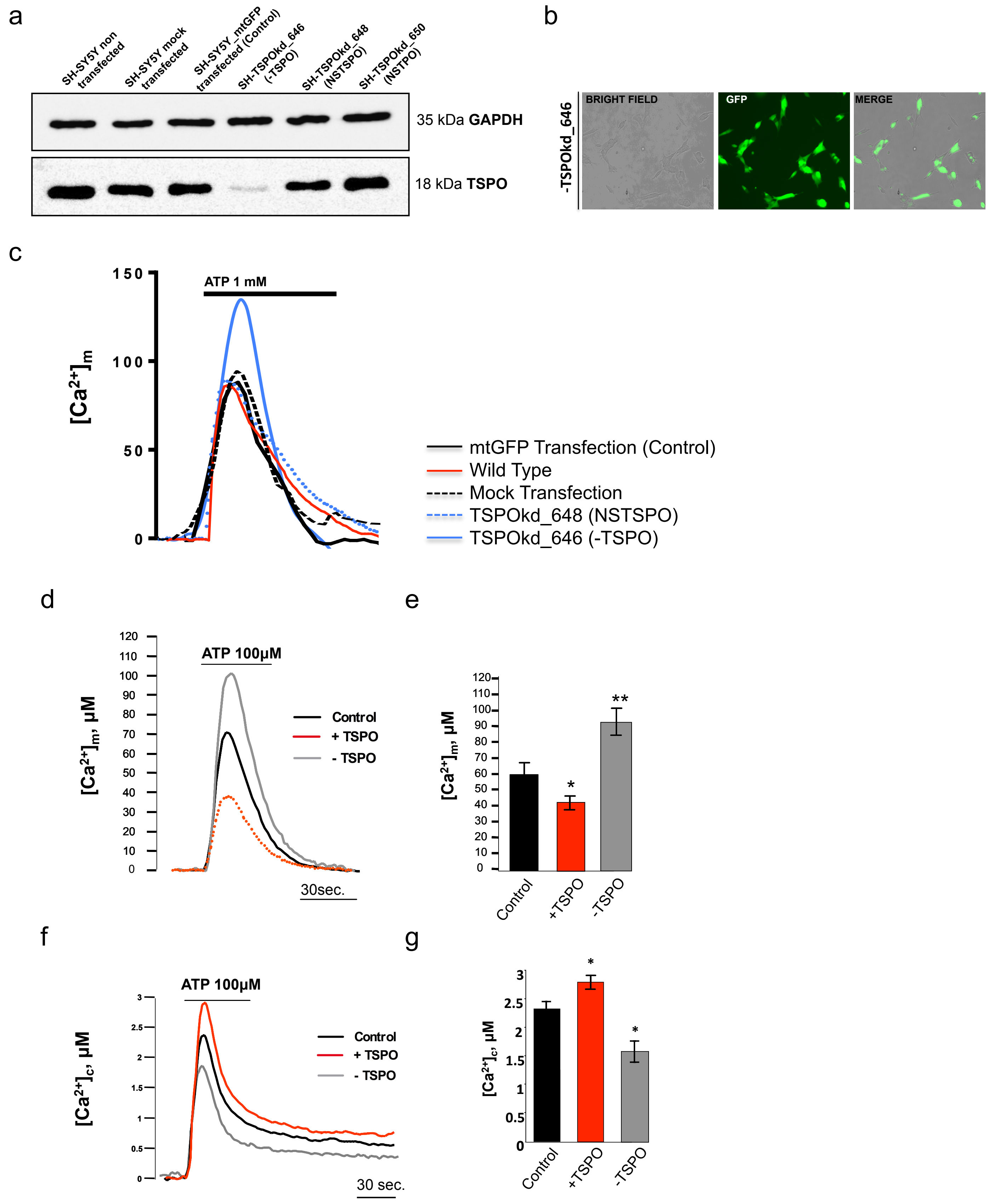
a
SH-SY5Y non transfected
SH-SY5Y mock transfected
SH-SY5Y_mtGFP transfected (Control)
SH-TSPOkd_646 (-TSPO)
SH-TSPOkd_648 (NSTSPO)
SH-TSPOkd_650 (NSTPO)
35 kDa GAPDH
18 kDa TSPO
b
-TSPOkd_646
BRIGHT FIELD
GFP
MERGE
c
ATP 1 mM
[Ca2+]m
150
100
50
0
mtGFP Transfection (Control)
Wild Type
Mock Transfection
TSPOkd_648 (NSTSPO)
TSPOkd_646 (-TSPO)
d
ATP 100μM
[Ca2+]m, μM
Control
+ TSPO
- TSPO
30sec.
e
[Ca2+]m, μM
Control
+TSPO
-TSPO
f
ATP 100μM
[Ca2+]c, μM
Control
+ TSPO
- TSPO
30 sec.
g
[Ca2+]c, μM
Control
+TSPO
-TSPO

Suppl. Fig. 2

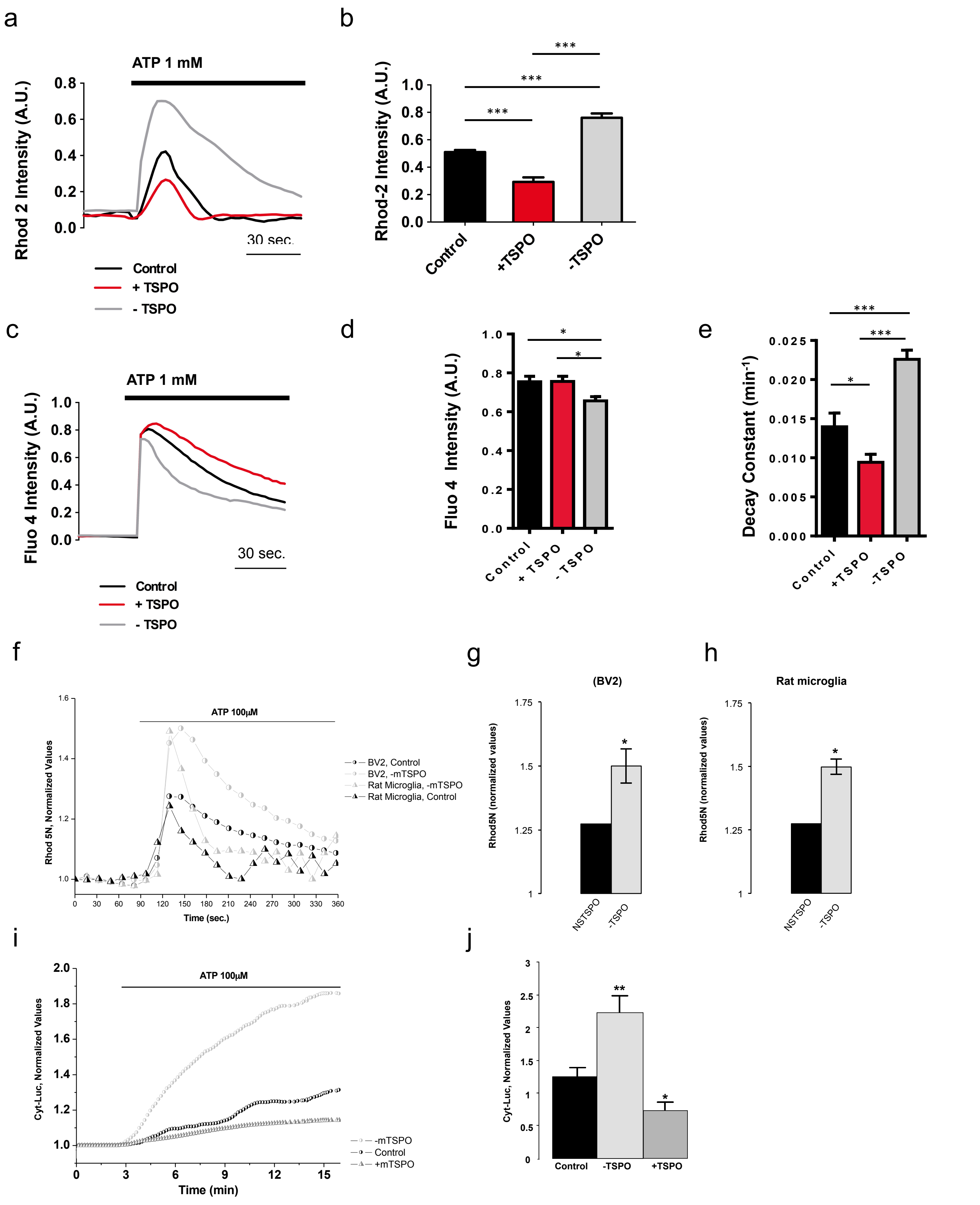

a
ATP 1 mM
Rhod 2 Intensity (A.U.)
0.8
0.6
0.4
0.2
0.0
30 sec.
Control
+ TSPO
- TSPO
b
Rhod-2 Intensity (A.U.)
1.0
0.8
0.6
0.4
0.2
0.0
***
***
***
Control
+TSPO
-TSPO
c
ATP 1 mM
Fluo 4 Intensity (A.U.)
1.0
0.8
0.6
0.4
0.2
0.0
30 sec.
Control
+ TSPO
- TSPO
d
Fluo 4 Intensity (A.U.)
1.0
0.8
0.6
0.4
0.2
0.0
*
*
Control
+ TSPO
- TSPO
e
Decay Constant (min-1)
0.025
0.020
0.015
0.010
0.005
0.000
***
***
*
Control
+TSPO
-TSPO
f
ATP 100μM
Rhod 5N, Normalized Values
1.6
1.4
1.2
1.0
0
30
60
90
120
150
180
210
240
270
300
330
360
Time (sec.)
BV2, Control
BV2, -mTSPO
Rat Microglia, -mTSPO
Rat Microglia, Control
g
(BV2)
Rhod5N (normalized values)
1.75
1.5
1.25
1
*
NSTSPO
-TSPO
h
Rat microglia
Rhod5N (normalized values)
1.75
1.5
1.25
1
*
NSTSPO
-TSPO
i
ATP 100μM
Cyt-Luc, Normalized Values
2.0
1.8
1.6
1.4
1.2
1.0
0
3
6
9
12
15
Time (min)
-mTSPO
Control
+mTSPO
j
Cyt-Luc, Normalized Values
3
2.5
2
1.5
1
0.5
0
**
*
Control
-TSPO
+TSPO

Suppl. Fig. 3

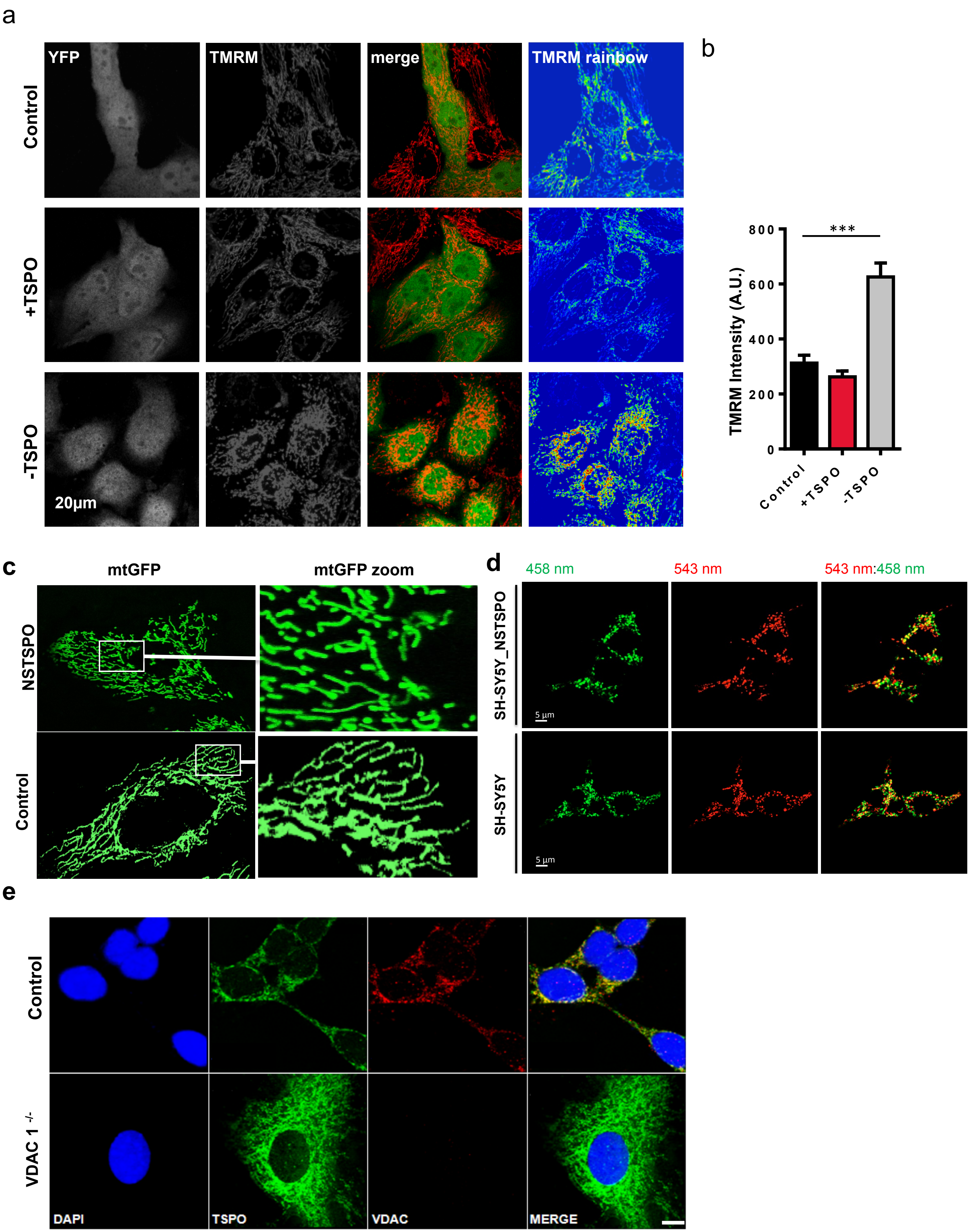
a
YFP
TMRM
merge
TMRM rainbow
Control
+TSPO
-TSPO
20µm
b
TMRM Intensity (A.U.)
800
600
400
200
0
***
Control
+TSPO
-TSPO
c
mtGFP
mtGFP zoom
NSTSPO
Control
d
458 nm
543 nm
543 nm:458 nm
SH-SY5Y_NSTSPO
SH-SY5Y
5 µm
5 µm
e
Control
VDAC 1 -/-
DAPI
TSPO
VDAC
MERGE

Suppl. Fig. 4

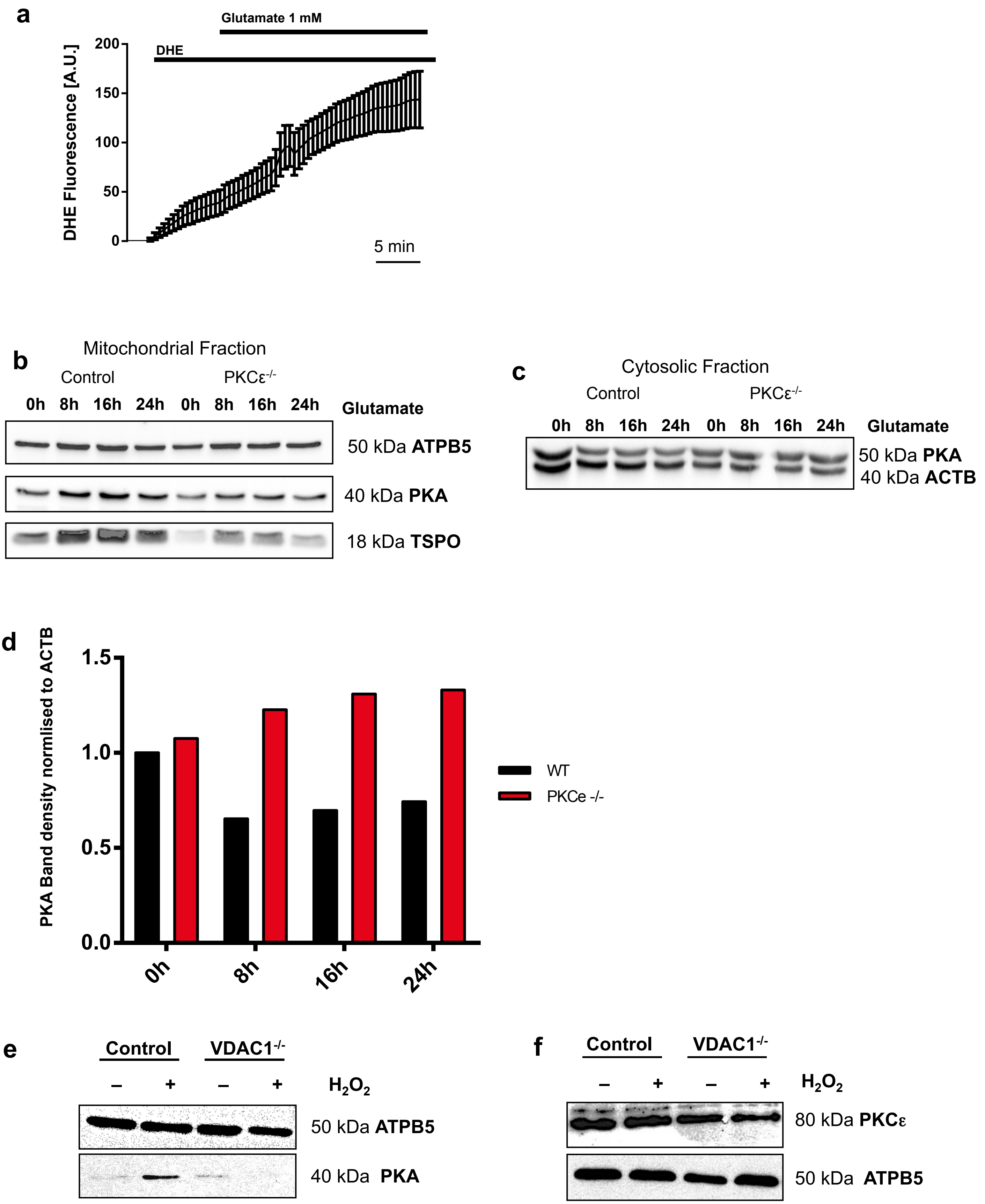
a
Glutamate 1 mM
DHE
DHE Fluorescence [A.U.]
200
150
100
50
0
5 min
b
Mitochondrial Fraction
Control
PKCε-/-
0h 8h 16h 24h 0h 8h 16h 24h
Glutamate
50 kDa ATPB5
40 kDa PKA
18 kDa TSPO
c
Cytosolic Fraction
Control
PKCε-/-
0h 8h 16h 24h 0h 8h 16h 24h
Glutamate
50 kDa PKA
40 kDa ACTB
d
PKA Band density normlised to ACTB
1.5
1.0
0.5
0.0
0h
8h
16h
24h
WT
PKCe -/-
e
Control
VDAC1-/-
– + – +
H2O2
50 kDa ATPB5
40 kDa PKA
f
Control
VDAC1-/-
– + – +
H2O2
80 kDa PKCε
50 kDa ATPB5

Legends to Figures

Supplementary Figure 1

(a) Western Blot reporting TSPO expression level in all experimental conditions including EmptyVector mtGFP which acted as Control, Mock Transfection, Silencing and Non Silencing (Scramble) shRNAs forTSPO. (b) Representative images of SH-SY5Y cells expressing GFP labeled TSPO_646 shRNA (-TSPO). (c) Representative recordings of Ca^{2+} transients ATP elicited in MEFs resembling the experimental conditions of TSPO modulated expression and relative controls. (d) Representative Ca^{2+} transients from HeLa cells transfected with mtAeq and stimulated with ATP (100 µM). (e) Graph showing the mean max $[Ca^{2+}]_m$ ($[Ca^{2+}]$max Control (mtGFP expressing cells): 58 ±11, +TSPO: 41±8, -TSPO:90±12 n=9; *$p<0.05$. (f) Representative Ca^{2+} transients from HeLa cells transfected with cyt-Aeq and stimulated with ATP (100 µM). (g) Graph showing the mean max $[Ca^{2+}]_c$ ($[Ca^{2+}]_{max}$, Control (mtGFP expressing cells): 2.32 ±0.35, +TSPO: 2.9±0.41, -TSPO:1.6±0.68, n=11; *$p<0.05$).

Supplementary Figure 2

(a) Representative mitochondrial Ca^{2+} traces obtained in CF38 cells loaded with Rhod-2 and stimulated with ATP (1 mM). (b) Graph showing the mean maximum Rhod-2 fluorescence intensity following application of ATP in CF38 cells. $p<0.001$; n>5 cells (from four independent experiments). (c) Representative cytosolic Ca^{2+} traces obtained in CF38 cells loaded with Fluo4 and stimulated with ATP (1 mM) (d) Graph showing mean maximum Fluo-4 fluorescence intensity in CF38 cells. n>17 cells; $p<0.05$. (e) Graph showing the mean rate of decrease in the Fluo-4 signal intensity after the ATP-induced peak in fluorescence [decay constant (min-1)]. $p<0.05$, $p<0.001$; n>17 cells (from three independent experiments). (f) Mitochondrial Ca^{2+} uptake (measured with Rhod5N in response to IP_3 generating stimulus ATP (100 µM), in microglia cell line (BV2) and primary cultures isolated from rat cortex following transient suppression of TSPO (-TSPO) quantified in (g) BV2 and (h) rat microglia, n=3; $p<0.05$. (i) Intracellular ATP analysis in HeLa cell cells using cytosolic luciferase, quantified in (j) n=5, $p<0.05$; $p<0.01$. Control conditions in Rhodamine and Luciferase based analyses were provided by mtGFP expressing cells, whilst for the Fluo-4 measurements the mtRFP was employed as Empty Vector Control.

Supplementary Figure 3

(a) Representative images of CF35 cells transfected with YFP and loaded with TMRM. A TMRM rainbow intensity panel is included to better visualize differences in intensity. A shift from blue colours to red-orange corresponds to an increase in TMRM fluorescence intensity. In –TSPO YFP-positive cells, there is a greater shift towards the red-orange colour range compared to Control (YFP expressing cells); in +TSPO YFP-positive cells TMRM labeling is lower thus remains in the blue colour range. (b) Mean maximum fluorescence intensity in CF38 cells; n>15; $p<0.001$. (c) Representative mid-plane cross-sectional images of the cellular mitochondrial network in SH-SY5Y control transfected with mtAeq and TSPO_648 (NSTSPO) cells, obtained through confocal microscopy (mtGFP) are depicted in the left panel. White boxes show regions of the cell magnified in the right sided panel. (d) Electromicrographs showing ER-mitochondria adjacency in SH-SY5Y and TSPO_648 (NSTSPO) transfected cells. (e) Immunofluorescence of WT and $VDAC1^{-/-}$ MEFs stained for TSPO (green) and VDAC (red).

Supplementary Figure 4

(a) Representative dihydroethidium (DHE) traces in SH-SY5Y cells treated with 1mM glutamate (n = 20; $p<0.05$). (b-c) Representative immunoblot of mitochondrial (b) and cytosolic (c) fractions isolated from $PKC\varepsilon^{-/-}$ MEFs and relative Control MEFs treated with Glutamate (20 mM) for 0-24 hours. (d) Quantification of cytosolic distribution of PKA over the duration of glutamate treatment, normalized to ACTB. (e) Western blot showing Protein Kinase A in mitochondrial fractions of $VDAC1^{-/-}$ MEFs and relative Control MEFs exposed to oxidative stress - 1 mM H_2O_2 for 1 hour. (f) Western blot showing protein kinase C epsilon (PKCε) in mitochondrial fractions of $VDAC1^{-/-}$ MEFs and relative Control MEFs exposed to oxidative stress - 1 mM H_2O_2 for 1 hour.
